# Inventory of state workers’ compensation laws in the United States: first responder mental health

**DOI:** 10.1057/s41271-024-00501-5

**Published:** 2024-07-12

**Authors:** Sherry Brandt-Rauf, Andrea L. Davis, Jennifer A. Taylor

**Affiliations:** Department of Environmental & Occupational Health, Dornsife School of Public Health at Drexel University, Philadelphia, PA USA

**Keywords:** Mental health, First responders, Policy

## Abstract

We conducted a comprehensive review of state workers’ compensation laws in the United States to evaluate the extent to which they support first responders with mental injury. Most state workers’ compensation systems divide mental injuries into categories based on their presumed etiology: physical-mental, mental-physical, and mental-mental. Major differences exist among states as to which workers are eligible. Proving workplace causation can be difficult where no traumatic physical injuries exist. Latency periods, time limits, preexisting health conditions, restrictions as to types of condition covered, and complex chains of causation may make this burden, which falls on the claimant, even more challenging. Only nine (9) states enacted presumption of causation laws for mental health conditions to ease claimants’ burden of proof. This contrasts starkly with presumption laws for chronic and infectious diseases. State decision-makers should create presumptions that mental health conditions in first responders are caused or significantly exacerbated by their stressful workplaces.

**Supplementary Information:**

The online version contains supplementary material available at 10.1057/s41271-024-00501-5.

## Key message

1. Workers’ compensation benefits for first responder mental health fall short compared to benefits for physical injury, and chronic and infectious disease.

2. State policies need to change to follow the growth in our knowledge of how common mental injury is among first responders.

3. Compassionate and expedient reimbursement of workers’ compensation claims for mental injury constitutes the fulfillment of our moral obligation to those who provide a critical public health safety net.

**Supplementary Information:**

The online version contains supplementary material available at 10.1057/s41271-024-00501-5.

## Introduction

**Fig. 1:**
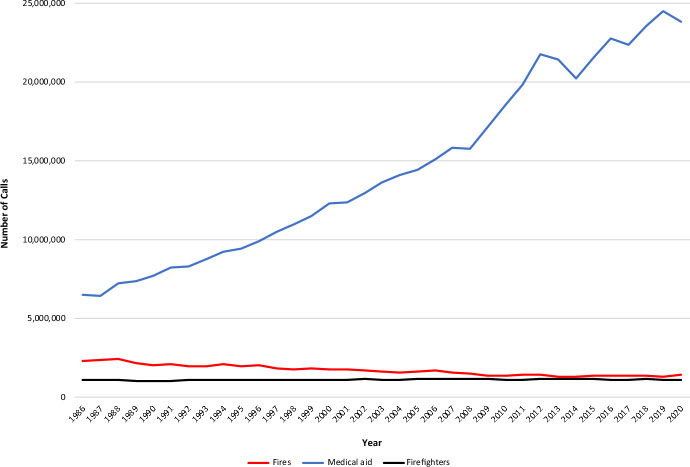
NFPA Survey of Fire Departments for US Fire Experience (1986–2020)

First responders face a considerable amount of stress on the job and witness traumatic events regularly [1–3]. The term ‘first responder’ refers to multiple professions. We use the term to refer to fire-based emergency medical service (EMS) personnel and firefighters. In 2021, the National Fire Protection Agency recorded 26 million EMS calls to fire departments, a 10% increase from 2020. As shown in Fig. 1, from 1980 to 2021, the number of EMS calls increased 421%, but the number of firefighters remained constant across that interval at approximately 1,040,000 [4].

Compounded by the stress engendered by this steep increase in call volume, exposure to stress and trauma on the job can lead to the development of psychological conditions including anxiety, depression, and post-traumatic stress disorder (PTSD). High-stress jobs have been long known to be associated with psychological strain, poor organizational outcomes, and physical illness [5–7]. Stress and trauma may compromise the critical risk assessment skills necessary for decision-making [5–7]. In the fire service, emotional exhaustion and low job engagement have been shown to be higher in EMS responders compared to their firefighter colleagues [8, 9]. Moreover, EMS responders experience higher rates of depression when emotional exhaustion is high [10]. According to a comprehensive report by the Substance Abuse and Mental Health Services Administration (SAMHSA), an estimated 30 percent of all first responders in the United States develop behavioral health conditions due to stressors on the job. This results in rates of depression, PTSD, and suicidality exceed those of the general population [1]. Thus, many first responders will pursue workers’ compensation or other disability payments.

Workers’ compensation is a no-fault system that replaces the fault-based tort law system that would otherwise compensate workers “who are injured or made ill in the course of employment…” [11]. With some exceptions, workers’ compensation is a matter of state law, and each state defines its coverage differently. Each state system contains numerous exemptions and exceptions that complicate a claimant’s process to gain compensation. States do not cover all illnesses or types of illnesses, nor do the laws and regulations consider all workers present in the workplace to be employees. According to the Social Security Administration, the federal governments originally established workers’ compensation in the early 1900’s for its employees engaged in dangerous work [12]. Several states followed in by the 1920s most states had workers compensation laws. Currently all 50 states and territories have workers compensation programs.

An issue that often generates questions about eligibility arises when the harm sustained by the worker is caused by an illness rather than an injury. Another occurs when the harm is psychological or emotional rather than physical. Claimants in these cases may find their claims barred or have difficulty meeting their burden of proving that the harm they sustained was sufficiently job-related. Long latency periods for some occupational diseases, as well as restrictions in some states against compensation for “ordinary diseases of life” complicate this task [13]. In Minnesota, “ordinary diseases of life to which the general public is equally exposed outside of employment are not compensable … Except where the exposure is peculiar to the occupation” [14]. Some states may foreclose or circumscribe recovery for psychological or emotional harm irrespective of cause.

Employees usually bear the initial burden of proving that their job caused the injury or illness. Some states have eased this often-substantial burden by creating ‘presumptions’ of workplace causation. Historically, states have established presumptions for individuals who have sustained harm through no fault of their own, and even through no fault of their employer [15]. Such presumptions can satisfy the employee’s initial burden of proof, transferring the burden to the employer to disprove the existence of workplace causation. In most states, these presumptions are ‘rebuttable’, meaning that the employer may introduce evidence that the injury or illness was not work-related, which may transfer the burden of proof back to the worker. Many states have presumption of causation laws specific to first responders for conditions including cardiovascular disease, respiratory diseases, certain cancers, and infectious diseases [16].

We began our research in June of 2020, by which point many EMS personnel across the country had adopted enhanced COVID-19 PPE (Personal Protective Equipment) protocols in response to growing concerns for first responder safety [17]. Some states added COVID-19 as the subject of presumption laws for first responders and healthcare providers, thus providing an opportunity for a natural comparison [of what?] with mental health.

### Study objectives

We sought to understand the ways in which first responder mental health state workers’ compensation laws addressed mental health. Our goal was to create an inventory of state workers’ compensation laws for mental health conditions and to compare the relevant attributes and language of these statutes.

## Data and methods

The research team used the Westlaw database to review each state’s workers’ compensation laws. We extracted and compiled relevant dimensions of the law into a word document and the attorney team member (SBR) reviewed them. These dimensions included definitions (employee, emergency service worker, paramedic, firefighter, injury, occupational disease, psychiatric and/or mental injury); time limits (to notify employer, to file claim with workers’ compensation board); and qualifying standards for workers’ compensation coverage involving mental health. The attorney team member resolved any discrepancies. We also identified presumptions of coverage for mental health conditions, whether they were part of the workers’ compensation program or other state programs including death and disability benefits, cancer funds, or others accessible to first responders. We looked at the language of the laws and cases indicating how courts have interpreted them. We compared presumption laws for mental health conditions to presumption laws in force for other conditions, including respiratory diseases, cancer, and infectious diseases. These data are current through December 2022.

## Results

### Presumptive laws

In Table 1, Part A we compare presumptions for mental injury with presumptions for other conditions. Cancer presumption is the most common (44 states), with cardiovascular disease (41 states), respiratory disease (39 states), and infectious disease (28 states) following.

**Table 1:**
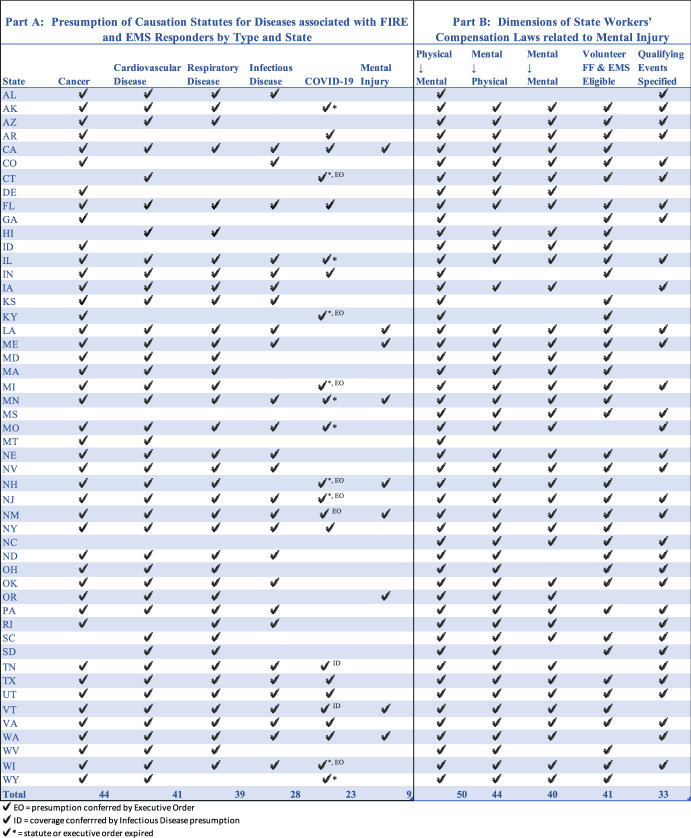
Presumption of Causation Statutes for Diseases associated with FIRE and EMS Responders by Type and State (Part A), and Dimensions of State Workers’ Compensation Laws related to Mental Injury (Part B).

Nine (9) state workers’ compensation systems include rebuttable presumptions of causation under certain circumstances for mental injury. Created between 2010 (Oregon) and 2020 (California), there is considerable variation in the language used and in qualifying factors claimants must establish for entitlement to the presumption. Maine, for example, does not set a minimum length of employment for eligibility, but does require the circumstances surrounding the onset of the occupational disease to have involved abnormal or unexpected stressors. Maine uses the term “average employee” as the comparison group for working conditions [18], while other states with presumptions of causation use the “general public” as the comparison group. Washington requires at least ten years of employment before a first responder-workers’ compensation claimant can use the PTSD presumption [19], but it does not specify the need for abnormal conditions.

**Fig. 2:**
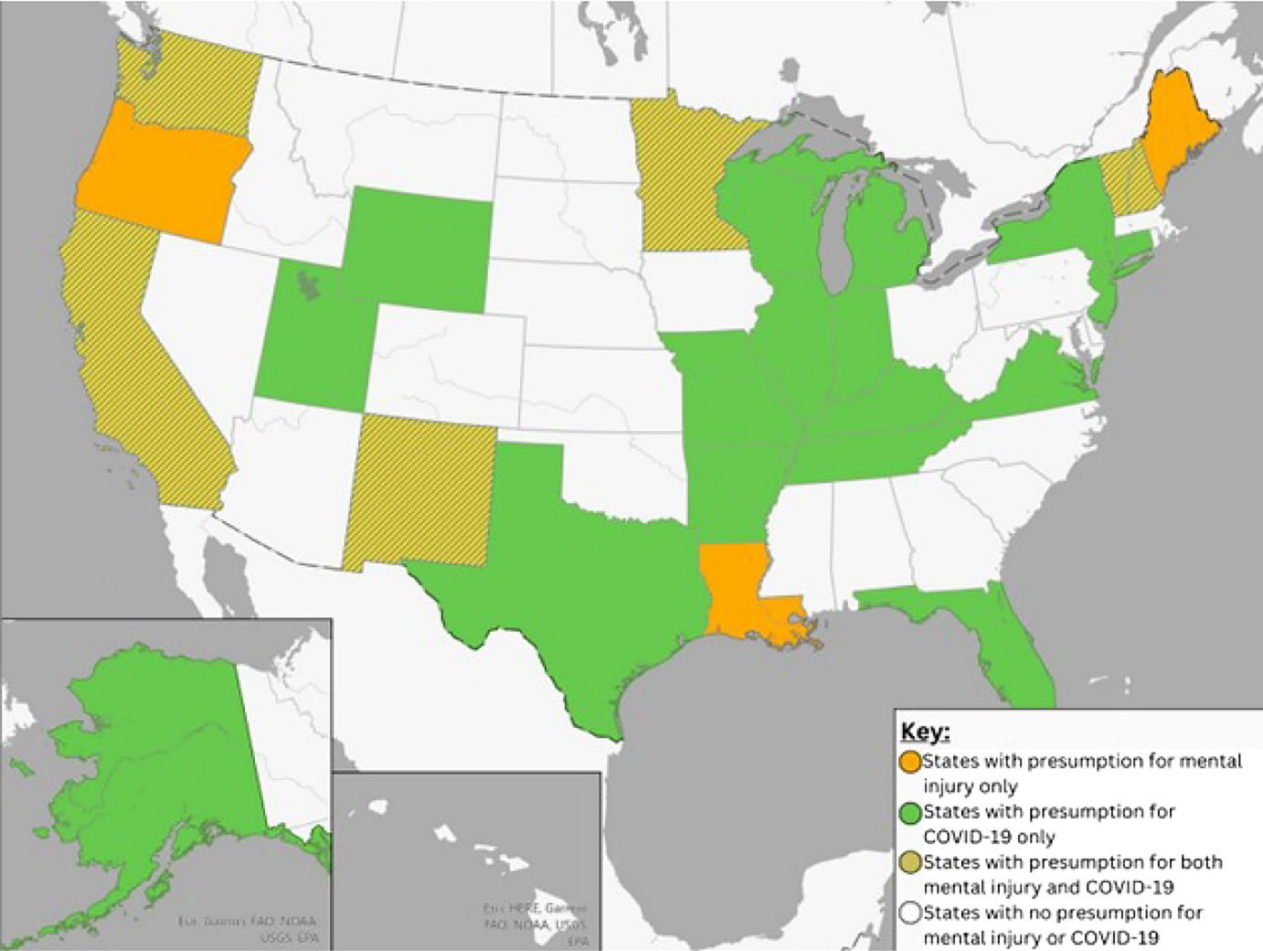
States with Presumption Laws for Mental Injury and COVID-19

During our research, some states added COVID-19 as a subject of presumption laws for first responders and health care providers. As such, it presented an opportunity to compare states by which enacted laws dealing with mental health (Fig. 2). Twenty-three (23) states created such presumptions. The populations to which these presumptions applied varied but typically included frontline health workers, essential employees, and first responders. In ten states, the governor created the presumption by executive order; in others states legislatures enacted presumptions, often related to enactment of broader COVID-19 legislation. Eleven (11) of these presumptions expired during the study.

### Workers' Compensation Mental Injury Categories by State

We found presumptive laws covering mental injury to be relatively rare, but benefits for mental injury through traditional workers’ compensation system to be very common. As with all workers’ compensation claimants, those seeking compensation for psychological injury must meet state standards of eligibility. These laws are not exclusive to firefighters and EMS responders; instead, these benefits extend to all workers to whom the compensation system applies. State workers’ compensation laws for mental injury divide claims into three categories based on the etiology of the condition. We follow each category with an example:

- Physical-to-mental injury (if an employee breaks a leg, develops depression from an inability to work);
- Mental-to-physical injury (if an employee is stressed because of work pressures, has a heart attack on the job);
- Mental-to-mental injury (if an employee faces stressful circumstances at work, either as a single incident or on an ongoing basis, which result in post-traumatic stress disorder).

Employees suffering from a physical-to-mental injury, where their mental health condition arose as a direct result of a workplace-related physical injury, are eligible for workers’ compensation in all states. Kentucky law specifies that an injury “shall include an occupational disease and damage to a prosthetic appliance, but shall not include a psychological, psychiatric, or stress-related change in the human organism, unless it is a direct result of a physical injury” [20]. Other states provide compensation even if the physical injury just contributed to the mental injury. Georgia case law provides that, “The physical injury need not be the precipitating cause of the psychic trauma; it is compensable if the physical injury contributes to the continuation of the psychic trauma” [21].

Employees suffering from mental-to-physical injuries, which are physical conditions that arise as a direct result of mental health stressors may be eligible for compensation in 44 states. Minnesota law states that, “Physical stimulus resulting in mental injury and mental stimulus resulting in physical injury shall remain compensable” [22]. Employees suffering from mental-to-mental injury**,** an injury involving purely mental stimuli, may be eligible for compensation in 40 states. In Montana, one of the states that does not provide such coverage, a firefighter was denied compensation for PTSD. The decision-makers did not regard his condition a physical-to-mental injury resulting from his burns, but as a mental-to-mental injury resulting from “emotional or mental stress surrounding a house-fire explosion” [23]. In all events, regardless of the nature and etiology of the injury, the other state requirements for compensation must be satisfied.

### Definitions

States’ determination of eligibility for workers’ compensation for mental health conditions depends on language used to define key terms. For example, some states cover volunteer firefighters: Pennsylvania provides definitive coverage [24]; Alabama [25] and Montana [26] statutes state that employers may opt to provide coverage. States also vary in how they characterize a mental health condition—as an occupational disease or an injury. Some use both classifications depending on the circumstances leading to the onset of the condition. Missouri law refers to “mental injury” [27]. California [28] and Vermont [29] classify mental health conditions as injuries; Oregon law states that PTSD or Acute Stress Disorder “shall be presumed to be compensable as an occupational disease” for covered employees, which include paid—but not volunteer—firefighters [30]. Time limits to provide notice of illness to the employer and to submit a claim may vary based on these classifications; some states provide more time for those with occupational diseases due to the latency periods associated with certain illnesses.

### Special coverage for first responders

Some states provide compensation for mental health conditions but only for certain populations, generally including first responders. Connecticut limits eligible claimants to “police officer, firefighter, emergency medical services personnel, Department of Correction employee, telecommunicator or health care provider” [31]. Texas provides that “post-traumatic stress disorder suffered by a first responder is a compensable injury…” under certain conditions [32]. States that have special coverage for first responders often fail to compensate for mental-to-mental injury for other employees (including Ohio, West Virgina; see Table 1).

### Employment conditions

States factor in employment conditions when determining eligibility for workers’ compensation. States often characterize employment conditions by comparing populations. In Illinois, case law holds that employees need only establish that “the employment conditions, when compared with the nonemployment conditions, were the major contributing cause of the mental disorder” [33]. Maine provides compensation for mental-to-mental injuries, but the claimant must demonstrate by clear and convincing evidence that, “The work stress was extraordinary and unusual in comparison to pressures and tensions experienced by the average employee” [34]. Some states do not specify comparison groups for working conditions, but predicate compensation on causative stressors that must be unexpected, or working conditions that must be abnormal for that type of employment. Pennsylvania case law requires claimants to prove that their “psychic injury was more than a mere subjective reaction to normal working conditions” [35]. In Alaska, coverage for mental injury is “not payable for mental injury caused by mental stress, unless it is established that (1) the work stress was extraordinary and unusual in comparison to pressures and tensions experienced by individuals in a comparable work environment; and (2) the work stress was the predominant cause of the mental injury” [36]. Similarly, in Arizona, “A mental injury, illness or condition shall not be considered a personal injury by accident arising out of and in the course of employment and is not compensable pursuant to this chapter unless some unexpected, unusual or extraordinary stress related to the employment or some physical injury related to the employment was a substantial contributing cause of the mental injury, illness or condition” [37]. Colorado law specifies that, “Accident”, “injury”, and “occupational disease” shall not be construed to include disability or death caused by or resulting from mental or emotional stress unless it is shown by competent evidence that such mental or emotional stress is proximately caused solely by hazards to which the worker would not have been equally exposed outside the employment [38]. Each of these critical terms—abnormal, extraordinary, unusual—is subject to interpretation by administrators and judges. What work conditions might be considered usual for first responders during a pandemic?

### Specified qualifying events required

Perhaps to avoid ambiguity inherent in terms like abnormal and unusual, some states allow first responders compensation for mental injuries only if the employee has witnessed a specified traumatic event. In Florida, the list includes, “Seeing for oneself a deceased minor” and “Directly witnessing an injury, including an attempted suicide … if the person was injured by grievous bodily harm of a nature that shocks the conscience” [39]. Connecticut lists six events, including witnessing a deceased minor [40]. Although this type of statute imparts less flexibility, it avoids difficulty for administrators and courts in identifying unusual work stress. This type of statute, however, ignores stress that can be experienced cumulatively by first responders when every day is stressful–despite absence of a single identifiable stressful event. What type of injury “shocks the conscience?”.

## Discussion

We found that all 50 states provided workers’ compensation coverage for physical-to-mental injuries, 44 states covered mental-to-physical injuries, and 40 states covered mental-to-mental injuries. It is a clear positive for workers that so many state systems cover mental-to-mental injuries. However, diminution in coverage as the type of injury moves from physical to mental is noteworthy. It may stem in part from the origins of workers’ compensation as a system in which claims for physical harm predominated.

We have shown that even states that cover mental injury in some cases treat claimants under this provision differently from those with physical injury and often place limitations on coverage that do not exist in the case of physical injury. As noted, some states limit compensation to those in particular occupations or who have experienced particular events. Other differences among state systems include coverage of volunteer firefighters, latency periods, time limits, the role of preexisting health conditions, restrictions as to type of condition covered, and the treatment of complex chains of causation. States differ in whether they consider mental harm an injury or a disease. The distinction may shape how a state deals with the mental health claims of first responders. Time limits to provide notices of illness to employers and to submit claims for compensation often vary based on this classification. The latency period of the onset of mental harm and the stigma often associated with it may affect the rapidity with which such claims can be filed and, as a result, the likelihood of a first responder to receive compensation. In Florida, even when mental injuries are compensable, there are limits on recovery if a physical injury is not also involved [41].

In cases that do not involve traumatic physical injury, proving workplace causation can be difficult. This challenging burden generally falls on the claimant. Some states have increased the burden in mental injury cases, raising the legal standard from ‘preponderance of the evidence’ to ‘clear and convincing evidence’ [42]. However, in recognition of this difficulty, and that mental injuries can be at least as disabling as physical injuries, if not more, nine states have enacted presumptions of workplace causation for mental health conditions. In these states, mental injury is, under specified conditions, presumed to arise from the workplace. For firefighters, presumptions bring mental injury in line with other conditions for which similar presumptions exist, among them certain cancers, respiratory illnesses, and infectious diseases. We do not know, however, the extent to which members of fire and rescue services are aware of these benefits. And even if they are aware, the likelihood of their submitting claims for mental injury may be affected by stigma as well as the notable barriers to coverage described. Future studies should explore this as well as the ways in which different types of claims may be treated differently. The enactment of progressive legislation related to mental health could do much to defeat the stigma among first responders connected with psychological harm.

Whether a state provides compensation for mental injuries reflects concerns about the cost and prevalence of mental health care and has implications far beyond the question of whether a particular worker is compensated for harm sustained in the workplace. The decision to link coverage for mental harms to physical conditions may reflect concerns about the potential for overwhelming the system financially if mental-mental claims are covered. This may be a particular issue in first responder workplaces in which virtually every worker is under extreme stress. It may even reflect romantic notions of first responders as invincible. State officials have expressed concern about the cost of compensating first responders for mental harms. The Montana legislature stated that, “a stress claim, often referred to as “mental-mental claims” or a “mental-physical claim”, is not compensable under Montana’s workers’ compensation and occupational disease laws. “The legislature recognizes that these claims are difficult to objectively verify and that the claims have a potential to place an economic burden on the workers’ compensation and occupational disease system” [43]. Failure to integrate mental and physical health may cost the workers’ compensation systems billions of dollars annually [44]. Failing to compensate workers for workplace-induced harm to their mental health can inflate medical costs, lead to increased absenteeism and resignations, and contribute to the stigma often attached to mental ill health. We can ill afford to have our first responders leave their jobs because of mental health-related disability. Supporting these workers with treatment while they remain in their jobs might well be a less costly alternative to losing them to total disability.

Rather than simply failing to compensate mental injury, following the COVID model, states could easily implement coverage for first responder mental illness as a statute with a built-in ‘sunset’ clause (such as termination date) as West Virginia has done [45]. Alternately, some states achieved rapid changes during COVID through executive order, often quicker and more agile than the lengthy legislative process often associated with enacting or updating statutes. Many more states have implemented COVID presumptions since the start of the pandemic in 2020 than have created presumptions for mental health in the last decade. Without such presumptions, it would have been very difficult for workers infected with COVID-19 to prove that they contracted the disease as a result of workplace exposures [46].

But perhaps more important than agility, causation, or cost, there is precedent in this country to protect people who put their lives at risk in service to others:

Presumptive disability laws applicable within the military developed in the 1920s, commencing in 1921 with the establishment of presumptions for neuropsychiatric disease and tuberculosis and the dissemination of the first publication of chronic diseases to which presumptions would apply. These early presumptions found their genesis in the exposure of service personnel that in short temporal intervals resulted in disease but which were neither measured nor classified at the time of exposure. Such early presumptive disability laws were grounded more on moral concern for returning veterans than scientific fact [47].

We have a moral duty to make first responders whole again after they serve for our communities. While presumptive laws are neither perfect nor uniform, they are an important tool in caring for the unique needs of first responders.

## Conclusions

State workers’ compensation systems today provide varying levels of support for first responders suffering from mental injury. Comparison shows important differences in coverage and eligibility. Given that first responders face extreme workplace stressors and trauma from their experiences in the workplace, more robust mental health presumptions for this occupational group would benefit first responders and support their mental health needs as they serve as our critical public health safety net.

### Supplementary Information

Below is the link to the electronic supplementary material.

Supplementary file1 (DOCX 24 KB)

## Data Availability

The authors confirm that all data generated or analysed during this study are included in this published article.
