# Supplementary material for: Inventory of state workers’ compensation laws in the United States: first responder mental health
Source: J Public Health Policy. 2024 Jul 12;45(3):562–74. doi: 10.1057/s41271-024-00501-5 (PMC11315667; doi:10.1057/s41271-024-00501-5)
Supplement: Supplementary file 1 — Supplementary file1 (DOCX 24 KB) [file 41271_2024_501_MOESM1_ESM.docx]

**State Presumption Laws**

- Alabama
  - Existing Presumptions
    - AL ST § 11-43-144. 2023. (hypertension, heart disease, respiratory disease, cancer, AIDS, hepatitis)
  - Mental Health Presumptions (none per Cocking)
    - Fruehauf Corp. v. Prater, 360 So.2d 999(Ala. Civ. App. 1978)]
    - Cocking v. City of Montgomery, 48 So. 3d 647, 650 (Ala. Civ. App. 2010)
- Alaska
  - Existing Presumptions
    - AK ST § 23-30-121 (2023).(respiratory disease, cardiovascular events, certain cancers)
  - Mental Health Presumptions
    - AK ST § 23-30-120(c) (2023). (No presumption of compensability for mental injury from work related stress).
- Arizona
  - Existing Presumptions
    - AZ ST § 23-901.09 & 23-1105 (2023). (certain cancers, cardiac, pulmonary)
  - Mental Health Presumptions (limited treatment, days off work)
    - AZ ST § 38-673 (2023).
- Arkansas
  - Existing Presumptions
    - AR ST § 21-4-107 (paid leave/cancer), 21-5-705 (Death benefit/cancer) (2023).
  - Mental Health Presumptions: Physical->Mental
    - AR Code § 11-9-113 (2023). (Mental illness not compensable unless caused by a physical injury except when caused by crime of violence).
  - Covid 19
    - Act 353 (H.B. 1488, 93^rd^ Gen. Assembly), 2021 Reg. Session (Ark. 2021). Expired 5/1/23.
- California
  - Existing Presumptions
    - CA LABOR § 4-1-3212.1 (2023)(cancer).
  - Mental Health Presumptions
    - CA LABOR Sec. 4-1-3212.15 (2023)
  - COVID-19 Presumption
    - CA LAB Sec. 3212.87 (expired 1/1/24)
- Colorado
  - Existing Presumptions
    - CO ST (§ 8-41-209 (2023) (cancer) & Sec. 8-41-208 (2023) (hepatitis C)
- Connecticut
  - Existing Presumptions
    - CT ST §§31-294i –31-294j) (2023)(cardiac; possibly hepatitis, meningitis, TB, Kahler’s disease, Lymphoma and certain cancers)
- Delaware
  - Existing Presumptions
    - DE ST Title 18 Secs. 6701 & 6701B (2023).18-6701B (2023)(firefighters with certain kind of cancer may recover from line of duty disability fund – no proof of causation necessary)
- Florida
  - Existing Presumptions
    - FL ST § 10-112.18 & 112.181(2023).(TB, heart disease, hypertension)(hepatitis, meningococcal meningitis or TB)
    - FL ST Sec. 10-112.1816 (2023) (alternative to workers compensation for certain cancers)
- Hawaii
  - Existing Presumptions
    - HI ST § 7-88-79 (2023). (for purposes of service-connected disability retirement coverage for disease of heart, lungs, or respiratory system resulting in permanent incapacity)
- Idaho
  - Existing Presumptions
    - ID ST § 72-438 (2023).(certain cancers)
- Illinois
  - Existing Presumptions
    - IL ST CH 820 Sec. 310/1 (2023). (disease from bloodborne pathogen, lung or respiratory disease, heart or vascular disease, hypertension, TB, cancer, hernia, hearing loss, covid)
  - Mental Health Presumptions
    - Pathfinder Co. V. Industrial Commission, 62 Ill.2d 556, 343 NE 2d 913 (1976).
    - Diaz v. Illinois Workers’ Compensation Commission, 2013 Il.App. 2d, 120294 WC (App. Ct. IL, 2d D. 2013).
    - Moran v. IL Workers’ Compensation Commission, 2016 WL 3579981 (IL App 1^st^ D. 2016)
    - Nw. Suburban Special Educ. Org. v. Indus. Comm'n, 312q Ill. App. 3d 783, 787, 728 N.E.2d 498, 501 (2000)
    - Weekley v. Indus. Comm'n, 245 Ill. App. 3d 863, 868, 615 N.E.2d 59, 63 (1993)
    - Baggett v. Indus. Comm'n, 201 Ill. 2d 187, 203, 775 N.E.2d 908, 917 (2002), as modified on denial of reh'g (Aug. 29, 2002)]
  - COVID-19 Presumption
    - IL ST CH 820 Sec. 310/1(g) (2023)
- Indiana
  - Existing Presumptions
    - IN ST § 5-10-15-9 (2023) (certain cancers, heart, lung disease, Parkinson’s)
    - IN ST Sec. 5-10-13-5 (2023) (infectious diseases including Covid)
    - IN ST Sec. 36-8-8-12.5 (2023) (related).
  - Mental Health Presumptions
    - Rayford v. Lumbermens Mut. Cas. Co., 851 F. Supp. 1243, 1246 (N.D. Ind. 1994)
    - Hansen v. Von Duprin, Inc., 507 N.E.2d 573 (Ind. 1987)
  - Covid Presumptions
    - IN ST Sec. 5-10-13-5 (2023).
- Iowa
  - Existing Presumptions
    - IA ST § 411.6 (2023). (retirement benefits) - not workers’ compensation (heart, lung, respiratory, cancer, infectious disease)
- Kansas
  - Existing Presumptions
    - KS ST§ 74-4952 (2023).(heart, cancer, bloodborne pathogen, lung) (retirement system)
- Kentucky
  - Existing Presumptions
    - KY ST §61.315 (2023) (death benefits for cancer)
  - COVID-19 Presumption
    - Executive Order 2020-277 (2020). https://governor.ky.gov/attachments/20200409_Executive-Order_2020-277_Workers-Compensation.pdf
- Louisiana
  - Existing Presumptions
    - LA RS 33:1948 (2023). (hepatitis B & C)
    - LA RS 33:2011 (2023). (cancer)
    - LA RS 33:2581, 2581.1 (2023)(lung disease, hearing loss).
  - Mental Health Presumptions
    - LA RS 33-2581.2 (2023).

Maine

- - Existing Presumptions
    - ME ST T. 39A Sec. 328 (2023). (heart, lung)
    - ME ST T. 39A Sec. 328A (2023). (hep, meningococcal meningitis, TB))
    - ME ST T. 39A Sec. 328B (2023). (certain cancers)
  - Mental Health Presumptions
    - ME ST T. 39A Sec. 201 (3-A)(2023)
- Maryland
  - Existing Presumptions
    - MD Labor and Employment Code Ann. § 9-503 (2023).(heart, hypertension, lung disease, some cancers)
- Massachusetts
  - Existing Presumptions
    - MA ST Sec. 94 (2023)(retirement systems and pensions)(hypertension, heart disease)
    - MA ST Sec. 94A. (2023) (retirement systems and pensions)(respiratory disease)
    - MA ST Sec. 94B (2023) (retirement systems and pensions)(cancer)
- Michigan
  - Existing Presumptions
    - MI ST 418.405 (presumed coverage fund: cancer, respiratory and heart diseases)(2023)
- Minnesota
  - Existing Presumptions
    - MN ST § 176.011 (2023) (myocarditis, coronary sclerosis, pneumonia, infectious disease, cancer, mental impairment, covid)
  - Mental Health Presumptions
    - MN ST § 176.011 (2023).
    - Smith v. Carver Cty., 931 N.W.2d 390, 398–99 (Minn. 2019)
  - Covid-19 Presumptions
    - MN ST § 176.011 (2023).
- Mississippi
  - Existing Presumptions
    - Mississippi First Responders Health and Safety Act, MS ST § 25-15-405 (2023). (an alternative to workers’ compensation for firefighters with certain types of cancer)
- Missouri
  - Existing Presumptions
    - MO ST § 87.006 (2023). (Infectious disease, lung disease, hypotension, hypertension, cardiac, cancer only for purposes of computing retirement benefits provided by an established retirement plan)
  - COVID-19 Presumption – expired
    - Would be covered by above
- Montana
  - Existing Presumptions
    - MT ST § 39-71-1401 (2023).(certain cancers, myocardial infarction)
- Nebraska
  - Existing Presumptions
    - NE ST § 35-1001 (2023).(cancer, blood borne infectious diseases, tuberculosis, meningococcal meningitis, MRSA)
    - NE ST § 18-1723 (2023). (Police Officers Retirement Act and pension
- Nevada
  - Existing Presumptions
    - NV ST § 617.453 (2023). (cancer)
    - NV ST Sec. 617.455 (2023). (lung disease)
    - NV ST § 617.457 (2023). (heart disease)
    - NV ST § 617.481 (2023). (infectious disease)
    - NV ST § 617.485 (2023). (hepatitis)
- New Hampshire
  - Existing Presumptions
    - NH ST § 281A-17 (2023). (heart and lung disease, cancer)
  - Mental Health Presumptions
    - NH ST § 281A-17c (2023).(acute stress disorder, PTSD)
- New Jersey
  - Existing Presumptions
    - NJ ST § 34:15-7.3 (2023). (cardiovascular, cerebrovascular)
    - NJ ST Sec. 34:15-43.2 (2023). (respiratory disease/volunteers)
  - COVID-19 Presumption
    - NJ ST Sec.34-15-31.12 (2023).
- New Mexico
  - Existing Presumptions
    - NM ST § 52-3-32.1 (2023).(cancer, hepatitis, TB, diptheria, meningococcal disease, MRSA)
  - Mental Health Presumptions
    - NMSA Sec. 52-3-32.1(B)(13) (2023). (PTSD)
  - COVID-19 Presumption
    - 2020 New Mexico Executive Order 20-025 (2020 NM EO 20-025)
    - <https://www.iaff.org/wp-content/uploads/NM-Executive-Order-2020-025.pdf> (through March 31, 2023). Accessed 1/23/24
- New York
  - Existing Presumptions
    - NY Gen Mun Laws § 207-k, kk, kkk, p, q (2023).(heart disease, stroke, cancer, Parkinson’s, HIV, TB, hepatitis, lung disease)
    - NY RET & SS §363-F (2023).(lung)
    - NY RET & SS § 363-FF (2023). (Parkinson’s)
  - COVID-19 Presumption
    - NY RET & SS Sec. 361(b)(2023) (Death benefit)
- North Carolina
  - Existing Presumptions
    - NC § Code 143-12A-166.2 (2023). (death benefits/cancer)
- North Dakota
  - Existing Presumptions
    - ND ST § 65-01-15.1, 15.2 (2023) (respiratory and heart disease, hypertension, disease caused by blood borne pathogen, cancer and, under some circumstances, cardiac event).
- Ohio
  - Existing Presumptions
    - OH ST § 4123.68 (w) & (x) (2023). (cardiovascular, pulmonary, respiratory, heart, blood borne disease, cancer)
  - Mental Health Presumptions
    - OH ST Sec. 126.65 (2023).(PTSD fund)
- Oklahoma
  - Existing Presumptions
    - OK ST T. 11 §49-110 (2023). (heart, respiratory, infectious disease and cancer0
- Oregon
  - Existing Presumptions
    - OR ST § 656.802(4) & (5)(2023).(lung, cardiovascular-renal disease, hypertension, cancer)
- Pennsylvania
  - Existing Presumptions
    - PA ST 77 P.S. Sec. 1208 (2023) together with P.S. Sec. 413 & 414 (2023) (cancer, occupational disease)
- Rhode Island
  - Existing Presumptions
    - RI ST § 45-19-1-2 (2023), 45-19.1-3 (2023), 45-19.1-4 (2023) (cancer)(conclusive presumption).
    - RI Gen. Laws 23-28.36-4, 28.37-1 (2023). (infectious disease)
- South Carolina
  - Existing Presumptions
    - SC Code of Laws § 23-9-197 (2023).(Firefighter Cancer Health Care Benefit Plan)
    - SC Code of Laws § 42-11-30 (2023).(heart or respiratory disease proximate to fighting a fire)
- South Dakota
  - Existing Presumptions
    - SDLR § 9-16-3.3 (2023).( pension)
    - [SDLR § 9-16-45 (2023). (respiratory & heart disease, hypertension).
- Tennessee
  - Existing Presumptions
    - Tenn. Code Ann. § 7-51-201 (2023).(lung or heart disease, hypertension/compensation other than workers’ compensation)
    - Tenn Code Ann. § 7-51-209 (2023).(infectious disease)
  - COVID-19 Presumption
    - Tenn Code Ann. § 7-51-209 (2023).(infectious disease)
- Texas
  - Existing Presumptions
    - TX GOVT § 6-607-051 (2021).
    - TX GOVT Sec. 6-607-054 (2023) (tuberculosis or other lung disease other than Covid)
    - TX GOVT Sec. 6-607.055 (2023) (cancer)
    - TX GOVT Sec. 6-607.056 (2023) (myocardial infarction, stroke)
    - TX GOVT Sec. 6-607.057 (2023) & 6-607.058 (2023) .

Covid Presumption

Texas Labor Code section 6-607.0545 (2023). (expired)

- Utah
  - Existing Presumptions
    - UT ST § 34A-2-901 (2023) with Sec. 78B-8-401 (infectious diseases inc. Covid)
    - UT ST § 34A-3-113 (2023). (cancer)
  - COVID-19 Presumption
    - UT ST § 34A-3-202 (2023) (covid) & Sec. 78B-8-401 (2023)(infectious disease).
- Vermont
  - Existing Presumptions
    - VT ST T.21 § 601 (2023).(heart injury or disease within 72 hours of service, cancer, lung or infectious disease))
  - Mental Health Presumptions
    - VT ST T.21 § 601 (2023). (PTSD)
  - COVID-19 Presumption
    - VT ST T.21 Sec. 601 (2023)
- Virginia
  - Existing Presumptions
    - VA ST § 65.2-402 (2023). (respiratory and heart disease, hypertension, cancer)
    - VA ST § 65.2-402.1 (2023). (hepatitis, meningococcal meningitis, TB, HIV)
  - Mental Health Presumptions
    - VA Code § 65.2-107 (2023).(presumption or indication of compensability).
  - COVID-19 Presumption
    - VA ST § 65.2-402.1 (2023).
- Washington
  - Existing Presumptions
    - WA ST § 51.32.185 (2023). (respiratory diseases, cancer, infectious disease, heart problems within 72 hours of firefighting)
  - Mental Health Presumptions
    - WA ST § 51-32-185 (2023). (PTSD)
  - COVID-19 Presumption
    - WA ST Sec. 51-32-185 (2023) (infectious diseases: list does not include Covid but see below)
    - <https://www.governor.wa.gov/news-media/inslee-announces-workers-compensation-coverage-include-quarantined-health-workersfirst> (including quarantined workers)
- West Virginia
  - Existing Presumptions
    - WV ST § 23-4-1 (2023). (cardiovascular or pulmonary disease, cardiovascular injury, cancer)
  - Mental Health Presumptions
    - WV Code Sec. 23-4-1f (2023) (PTSD under certain conditions)
- Wisconsin
  - Existing Presumptions
    - WI ST § 891-45 (2023).(heart or respiratory impairment or disease)(death or disability benefits, pension, retirement system)
    - WI ST § 891-453 (2023).(infectious disease)(same)
    - WI ST § 891-455 (2023).(cancer)(same)
  - COVID-19 Presumption
    - WI ST Sec. 891-453 (2023).
    - WI ST Sec. 102.03(6)(b) (2023)
- Wyoming
  - Existing Presumptions
    - WY ST Sec. 27-15-101 & 102 (2023). (cancer, cardiovascular disease, myocardial infarction , stroke and others “determined on a case by case basis”)
  - Mental Health Presumptions
    - WY ST Sec. 27-14-102 (a)(xi)(j) (2023).(mental injury compensable for first responders under certain circumstances).
    - WY ST § 27-15-101 (2023). (could include mental health)
  - COVID-19 Presumption
    - WY ST Sec. 27-14-102 (2023). (through 3/31/22 unless extended)
